# Male-Mediated Gene Flow in Patrilocal Primates

**DOI:** 10.1371/journal.pone.0021514

**Published:** 2011-07-01

**Authors:** Grit Schubert, Colin J. Stoneking, Mimi Arandjelovic, Christophe Boesch, Nadin Eckhardt, Gottfried Hohmann, Kevin Langergraber, Dieter Lukas, Linda Vigilant

**Affiliations:** 1 Department of Primatology, Max Planck Institute for Evolutionary Anthropology, Leipzig, Germany; 2 Junior Research Group Novel Zoonoses, Robert Koch Institute, Berlin, Germany; 3 Emmanuel College, University of Cambridge, Cambridge, United Kingdom; 4 Department of Anthropology, Boston University, Boston, Massachusetts, United States of America; 5 Large Animal Research Group, Department of Zoology, University of Cambridge, Cambridge, United Kingdom; Institut Pasteur, France

## Abstract

**Background:**

Many group–living species display strong sex biases in dispersal tendencies. However, gene flow mediated by apparently philopatric sex may still occur and potentially alters population structure. In our closest living evolutionary relatives, dispersal of adult males seems to be precluded by high levels of territoriality between males of different groups in chimpanzees, and has only been observed once in bonobos. Still, male–mediated gene flow might occur through rare events such as extra–group matings leading to extra–group paternity (EGP) and female secondary dispersal with offspring, but the extent of this gene flow has not yet been assessed.

**Methodology/Principal Findings:**

Using autosomal microsatellite genotyping of samples from multiple groups of wild western chimpanzees (*Pan troglodytes verus*) and bonobos (*Pan paniscus*), we found low genetic differentiation among groups for both males and females. Characterization of Y–chromosome microsatellites revealed levels of genetic differentiation between groups in bonobos almost as high as those reported previously in eastern chimpanzees, but lower levels of differentiation in western chimpanzees. By using simulations to evaluate the patterns of Y–chromosomal variation expected under realistic assumptions of group size, mutation rate and reproductive skew, we demonstrate that the observed presence of multiple and highly divergent Y–haplotypes within western chimpanzee and bonobo groups is best explained by successful male–mediated gene flow.

**Conclusions/Significance:**

The similarity of inferred rates of male–mediated gene flow and published rates of EGP in western chimpanzees suggests this is the most likely mechanism of male–mediated gene flow in this subspecies. In bonobos more data are needed to refine the estimated rate of gene flow. Our findings suggest that dispersal patterns in these closely related species, and particularly for the chimpanzee subspecies, are more variable than previously appreciated. This is consistent with growing recognition of extensive behavioral variation in chimpanzees and bonobos.

## Introduction

Dispersal, the shift in residence of an individual from one area or social group to another, is a fundamental process affecting population structure. It is typically considered an inbreeding avoidance mechanism [1] and sex biases in dispersal appear to be influenced by sex–specific costs of local competition and dispersal [2], [3]. In most mammalian species males disperse, which potentially allows females to retain life–long residence in their natal area and gain indirect fitness benefits by cooperating with same–sex kin [4]–[6]. The less typical pattern of females dispersing and males remaining in their natal groups is also seen, for example, in some bats [greater spear–nosed bat; 7] and shrews [greater white–toothed shrew; 8], as well as in several primate taxa (muriquis [9]; spider monkeys [10], [11]; woolly monkeys [12]; hamadryas baboons [13]; red colobus monkeys [14]; bonobos [15]; chimpanzees [16]; many extant human populations [17]). The extent to which male movement or the transmission of male genes between social groups is limited by male philopatry in chimpanzees and bonobos is of interest, given the long–standing suggestion that male philopatry is an important trait shared by chimpanzees, bonobos and early humans and may have played a role in the evolution of affiliative and cooperative behaviors in these taxa [18], [19].

Dispersal is an intrinsically infrequent event that is seldom observed, particularly for species with slow life histories such as primates, and may be difficult to distinguish from the disappearance or death of individuals. Advances in the use of non–invasive samples to genetically characterize individuals and in the analysis of genetic data offer an opportunity to investigate this important, often cryptic aspect of life history in wild animal populations [20], [21]. Patterns of genetic variation estimated using biparentally–transmitted markers have been used in many species to confirm or reveal sex biases in dispersal tendencies or distances (e.g. common vole [22]; European roe deer [23]; greater white–toothed shrew [8]; mountain gorilla [24]; woolly and spider monkeys [25]). Moreover, sex–specific markers like the maternally–transmitted mitochondrial genome (mtDNA), the paternally–transmitted Y–chromosome or the predominantly female transmitted X–chromosome allow an explicit assessment of dispersal patterns for each sex (e.g. gray mouse lemur [26]; rhesus macaque [27]; reviewed for humans in [28]), while a combination of different marker systems seems a particularly useful approach [13], [26], [29].

There are many mechanisms by which the putatively nondispersing sex may contribute to gene flow between groups. In most taxa, the dispersal bias between the sexes is observed to be strong but not absolute, and some adult individuals of the typically philopatric sex may successfully join new groups (e.g. capuchin monkey [30]; greater sac–winged bat [31]). Infrequent events such as group dissolutions may distribute individuals into new groups, and this ‘involuntary’ form of dispersal may be difficult to distinguish from natal dispersal from genetic evidence alone (e.g. Belding's ground squirrel [32]; black and white colobus monkey [33]; savannah baboon [34]).

In addition, gene flow may take place even in the absence of dispersal by adult individuals of the philopatric sex. For example, in a male–philopatric species, breeding–age females may undergo secondary dispersal events and be accompanied by dependent male offspring. Furthermore, gene flow between groups may potentially take place in the absence of physical dispersal via copulations during intergroup encounters. In many birds and some mammals, incidents of extra–pair or extra–group paternity are frequent [35], potentially leading to discrepancies between the social and genetic structure of the population. Extra–group paternity (EGP) is also well–documented in primates including chimpanzees and bonobos [summarized in 36].

Intense aggression between adult males of different social groups would seem to effectively prevent adult male dispersal in chimpanzees [37]–[41], but bonobos appear to lack such extreme intergroup hostility among males [42]–[45]. Secondary dispersal of females accompanied by male offspring appears infrequent, but has been observed in cases of group dissolutions in eastern chimpanzees [46] or for unknown reasons in both species [47,48; Hohmann, unpublished data]. However, young immigrants would appear to face high risks of infanticide by resident males and even females, at least in chimpanzees [reviewed in, 49,50].

Facilitated by the fission–fusion nature of *Pan* society in which individuals often range in small subgroups or even alone [15], [51]–[53], solitary females might encounter and copulate with males from other groups. The circumstances surrounding extra–group copulations are best documented in western chimpanzees and feature both apparent male coercion and female choice, with observations of females detained by extra–group males and on other occasions paying short–term, apparently voluntary visits to neighbouring groups [41], [48]. Genetic analyses have shown that up to 10% of the offspring born in some western chimpanzee groups, and 5% in one bonobo group are sired by fathers from outside the mother's group [54]–[56], whereas extra–group conceptions are not known in the eastern chimpanzee subspecies [57,58; K. Langergraber, unpublished data]. In sum, the observational evidence suggests that while adult male dispersal is apparently uncommon, male–mediated gene flow through other sources potentially affects population genetic structure in these highly patrilocal species and might vary among different populations or subspecies.

Previous studies of patterns of differentiation for the maternally–transmitted mitochondrial DNA (mtDNA) and paternally–transmitted Y–chromosome in eastern chimpanzees and bonobos suggested that male–mediated gene flow is absent in eastern chimpanzees and potentially rare in bonobos [59]–[62]. However, these studies aimed at describing large scale patterns of genetic structure and hence employed samples collected over a wide geographic range. As one would predict that male–mediated gene flow, if present, occurs primarily among neighboring groups, fine–scaled sampling of adjacent groups in contiguous habitats is essential for investigating the incidence of male–mediated gene flow in chimpanzees and bonobos. A recent study of autosomal genetic variation at such a local scale in three western chimpanzee groups did not detect significant differentiation among groups and found genetic differentiation for males to be only slightly higher than for females [63]. However, how limited genetic differentiation at autosomal loci relates to differentiation at the Y–chromosome, and how it changes over different geographical scales, has not been investigated in *Pan*. Thus, analysis of both autosomal as well as Y–chromosomal loci seems necessary to fully understand dispersal patterns in the *Pan* species and subspecies.

In this study, we investigated potential differences in the amount of genetic differentiation and of male–mediated gene flow between wild western chimpanzees (*Pan troglodytes verus*) and bonobos (*Pan paniscus*). We used autosomal, as well as Y–chromosomal microsatellite markers to characterize genetic variation in multiple social groups. For investigation of genetic differentiation, in both species we applied a small scale sampling regime, and for the western chimpanzees also examined groups over a slightly larger geographical range to assess whether, as might be expected, genetic differentiation increases as geographic distance increases. Our goal was then to assess the evidence for male–mediated gene flow in these two male–philopatric primates. Potential sharing of Y–chromosomal variants is not necessarily evidence for male gene flow, but could also represent the retention of ancestral haplotypes in different social groups. We therefore employed a simulation approach to examine, under realistic estimates of relevant demographic factors (group size, male reproductive skew and mutation), expected levels of within–group Y–chromosomal genetic variation and to identify potentially immigrant types. By comparing our simulation results to our empirical data, we derived a potential range of levels of male–mediated gene flow in both study populations.

## Methods

### Study populations, sample collection and DNA extraction

**Figure 1:**
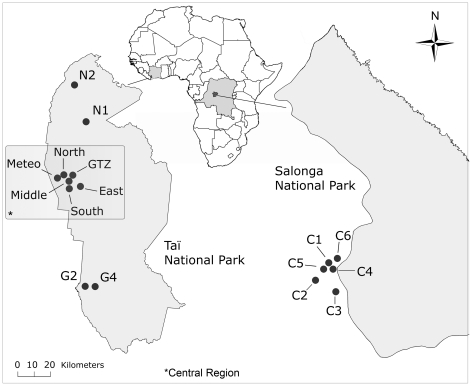
Geographical locations of genotyped individuals from social groups of western chimpanzees and bonobos. Western chimpanzee samples were collected within Taï National Park, Côte d'Ivoire, and bonobo samples at the border of Salonga National Park, DRC. Labels designate known (C1, East, Middle, North, South) and assumed (C2–6, G2, G4, GTZ, Meteo, N1– 2) social groups. The Central Region in Taï National Park represents a geographically limited subsample of chimpanzee groups analyzed in addition to the full data set (see also Table 1 and 2). Y-chromosomal data from bonobo group C3 were taken from [61], while autosomal data were not available for that group. Sample sizes of individuals with an estimated minimum age of 5 for each group are the following (females typed at autosomal loci/males typed at autosomal loci/males typed at Y-chromosomal loci): bonobo, C1 (17/11/10), C2 (14/10/15), C3 (0/0/6), C4 (12/9/6), C5 (4/3/2), C6 (8/3/3); chimpanzee, East (8/10/8), G2 (10/7/7), G4 (4/3/3), GTZ (3/8/6), Meteo (13/6/3), Middle (7/4/4), N1 (2/2/2), N2 (6/2/2), North (14/9/4), South (31/26/15).

We used the two–step ethanol–silica method [64] to collect noninvasive fecal samples from members of four habituated and six unhabituated groups of western chimpanzees in Taï National Park (TNP), Côte d'Ivoire [53; Figure 1]. A small proportion of chimpanzee fecal samples were simply dried on silica gel (N = 17) or frozen (N = 69). We collected bonobo samples from five neighboring groups, including one habituated research group [65], at the southwestern border of Salonga National Park, Democratic Republic of Congo (Figure 1). Samples from unidentified individuals were assigned the same social group when found together at a night nesting site, or when at least one individual was detected at multiple nest sites. Samples from unidentified individuals were assigned to different groups if the sampling locations were separated by at least 10 km [following, 61] or if the group's territory was known.

In total, 294 chimpanzee and 266 bonobo samples were collected and analyzed. We extracted all samples using the QIAamp DNA Stool kit (QIAGEN) with slight modifications [64]. DNA concentrations were estimated using a quantitative PCR assay [66]. DNA extracts from two–step ethanol–silica samples, silica samples and frozen samples contained on average 406±, 172±355 and 140±303 pg DNA/µl (mean ±1 SD), respectively. An additional 117 chimpanzee DNA extracts generated for previous studies [54], [55] were also used.

### Genotyping

Sex was determined or confirmed using polymerase chain reaction [67] amplification of a segment of the X–Y homologous amelogenin locus as previously described in detail [68]. We genotyped DNA extracts at 19 autosomal and 13 (chimpanzee) or 10 (bonobo) Y–chromosomal loci using a two–step amplification method as previously described [69; Information S1]. In brief, we combined either all autosomal [69] or Y–chromosomal primer pairs [70] with template DNA in an initial multiplex PCR reaction, then used dilutions of the resultant PCR products for amplification of each individual locus using fluorescently labeled forward primers and nested reverse primers in singleplex PCR reactions. For autosomal genotypes, at least three replicates were required to confirm homozygous genotypes with high confidence >99%, [69]. We accepted heterozygous autosomal genotypes after we observed each allele in at least two independent PCR reactions.

Because not all of the samples used came from habituated groups of individually identified animals, we used CERVUS 3.0 [71] to calculate *pIDsib*, the probability that two identical multi–locus genotypes do not come from the same individual but rather from siblings. Estimated average *pIDsib* values were <0.01 for our five most informative autosomal loci (chimpanzees and bonobos), or for the six (bonobo) or seven (chimpanzee) least variable loci, indicating that even partially complete genotypes from close relatives would be distinguishable (data not shown). Genotypes from different samples that were found to come from the same individual (*pIDsib* <0.01) were combined into a consensus genotype. In total we genotyped 203 individual chimpanzees (genotypes 97.0 % complete) and 101 bonobos (genotypes 92.8% complete) at 19 autosomal loci (see Table S1 for locus–specific characteristics). We also included 32 additional eight–locus chimpanzee genotypes generated previously [55] from individuals for which samples or extracts were no longer available, making the chimpanzee data 89.3% complete.

Each male was then genotyped at 13 (chimpanzee) or 10 (bonobo) Y–chromosomal loci developed in humans and previously assayed in eastern [62] and central chimpanzees [70] and bonobos [61]. To guard against false alleles when genotyping Y–chromosomal loci, for each locus an allele was confirmed if it was seen in two independent PCRs and no other allele was observed. We also included Y–haplotypes from an additional 11 bonobos typed previously [61] and originating from group C2 and an additional social group, C3 (Figure 1). Y–haplotypes were 99.5% (chimpanzee, N_Individuals_ = 87) and 97.2% (bonobo, N_Individuals_ = 47) complete.

Previous research on habituated bonobos and chimpanzees has shown that it is difficult to obtain samples from young individuals, particularly dependent infants [aged 0 – 4 years; 54]. Therefore, we assumed that our sample of unhabituated chimpanzees and bonobos would primarily consist of adult, adolescent and some juvenile individuals. To obtain comparable, approximately single–generation data sets from the habituated research groups, we used only genotypes from individuals present in 2001 and 2009 for the chimpanzees and bonobos, respectively, and the samples from unhabituated individuals were collected over a span of several months. We thereby excluded individuals that were known or estimated to be younger than 5 years.

### Genetic differentiation between social groups

We measured genetic differentiation F_ST_ between pairs of social groups at the autosomes, for males and females separately, and at Y–chromosomal loci using ARLEQUIN 3.11 [72]. The associated *p*–values of group pairwise F_ST_ were obtained from the permutation procedure implemented in ARLEQUIN. We also examined overall genetic differentiation in an AMOVA framework. Here, inspection of the overlap in the 95% confidence limits generated by bootstrapping genetic differentiation values obtained in a locus–by–locus AMOVA was used to evaluate whether (a) autosomal genetic differentiation between males and females was different within species and when comparing bonobos and chimpanzees and (b) genetic differentiation of Y–chromosomal loci differed between bonobo and chimpanzee males.

We examined genetic differentiation at two hierarchical levels in the more widespread chimpanzee sample to (a) obtain a data set readily comparable to the more local bonobo sampling and (b) examine whether the amount of genetic differentiation increased at the larger scale as might be predicted [73]. Initial performance of a matrix–correlation test did not reveal an isolation–by–distance pattern [73; data not shown], but with the limited number of social groups included here this test might not be very useful. F_ST_–based genetic differentiation measures are not entirely independent of the amount of genetic variation present within groups, with high levels of genetic variation potentially leading to lower F_ST_ estimates than low levels of variation [74], [75]. In some extreme cases, F_ST_ can therefore be biased and should be corrected. To allow for a meaningful comparison of genetic differentiation at the haploid Y–chromosome, where levels of variation are relatively low and slight differences between the species might be consequential for F_ST_ estimation, we also used a standardized measure of genetic differentiation for the Y–chromosomal data. Standardized F_ST_ expresses genetic differentiation as the maximum amount of genetic differentiation possible given the amount of within–group variation [74], [75]. We followed the procedures outlined in detail in [62] to calculate standardized genetic differentiation and determine the associated 95% confidence intervals.

To minimize stochasticity, for all analyses of genetic differentiation between communities we excluded social groups with fewer than four sampled individuals. We chose four because in some of the completely sampled habituated groups only four individuals of the respective category (males and females genotyped at autosomal loci; males genotyped at Y–chromosomal loci) were present.

### Modeling Y–chromosomal variation within social groups

There are two ways to investigate gene flow between social groups; one is to focus on variation shared between different groups, and the other is to look at the number and types of variants present within groups. We performed simulations (using Java, Sun Microsystems Inc. 1994–2009) to examine whether the Y–chromosomal variation present *within* empirical bonobo and chimpanzee groups could be generated through mutation or must originate externally through male–mediated gene flow. Starting groups of a fixed number of males, with all males possessing identical Y–haplotypes, were simulated over discrete generations in which the individuals of one generation were used as the fathers for the next generation. Starting the simulations with groups that contained only dissimilar, highly divergent haplotypes produced identical results, as most haplotypes were lost rapidly and new, similar ones were subsequently generated by mutation. Therefore, we only report results for starting groups with one haplotype. The following factors were included in the simulations:

*i) Mutation generates new haplotypes in groups.* Individual Y–haplotypes mutate with a probability derived as the product of mutation rate and the number of Y–chromosomal loci (13 and 10 loci in chimpanzees and bonobos, respectively) typed in our empirical data, following a stepwise mutation model. We used the most recent published human mutation rate (2.2×10^–3^ mutations/generation [76]). It was shown that this estimate is applicable to chimpanzees and bonobos [77], and information from 62 chimpanzee father–son pairs provides a similar estimate (6.46×10^–3^, data not shown). Analyses using both mutation rates produced similar results, so we report here the results based on the better–substantiated human mutation rate estimate.

*ii) Skewed reproduction removes haplotypes from groups*. Reproduction among *Pan* males is expected to be skewed over the short term with males of higher social dominance rank enjoying a reproductive advantage [e.g., 54,56]. However, over an entire generation skew should be lower, as individual opportunities to reproduce change [78]. Lifetime reproductive skew data are not available from chimpanzees or bonobos, nor for most mammals. Therefore, each individual's Y–haplotype is assigned a probability of being represented in the next generation according to models of lifetime reproductive success derived from the human hunter–gatherer Ache population [79; Table S2]. A log–function was fitted to the Ache data to adjust skew to the group sizes used here. The human lifetime reproductive success was slightly less skewed than the lowest short term skew levels observed among wild chimpanzee groups [57]. This is consistent with what might be expected for lifetime skew distributions in comparison to short term patterns within a species [80] and thus suggests that the use of these data is reasonable for chimpanzees.

*iii) Male–mediated gene flow adds new divergent haplotypes to groups*. In our model, male–mediated gene flow adds unique, infinitely distant (divergent) Y–haplotypes to the group at a specified constant rate. We chose to make the immigrant haplotypes highly divergent so that they can be distinguished from new haplotypes that arise via mutation and are highly similar to those already present. Since we thus consider any highly similar haplotypes as arising via mutation and highly divergent haplotypes as products of immigration, we may underestimate the rate of male migration that contributes haplotypes to the group similar to ones already there.

The habituated Taï chimpanzee groups contain nine reproductively active males on average [range 1–14, 41], as does the one bonobo research group investigated here. We therefore considered simulations of groups containing 5, 9, 10 and 15 males as realistic for the examined populations.

All simulations reached stability after less then 100 generations and we simulated 1000 identical starting groups of the above sizes for 1000 generations. In order to see how much haplotypic variation could arise and be maintained in these closed groups with no gene flow, we averaged generations 100–1000 to examine the proportions of groups containing variation (more than one Y–haplotype) and the maximum number of mutations typically (more than 1% of the observations) found between any different Y–haplotypes present in the same group. From this simulation we thus inferred how many different haplotypes might be found in a group and how different they would be from one another. We then used our empirical data to examine how frequently groups contained multiple haplotypes that were more different from one another than we would expect given these simulation results, representing potentially immigrant haplotypes. In a second step we incorporated male–mediated gene flow by adding unique, infinitely distant Y–haplotypes to the simulated groups at pre–defined rates and then examined which rates of male–mediated gene flow were compatible with the frequency with which we observed empirical groups to contain ‘immigrant’ haplotypes. The source code for the simulation is available as electronic Information S2.

## Results

### Genetic differentiation between social groups

Autosomal genetic differentiation F_ST_ was similarly low among male and female chimpanzees and bonobos (Table 1). While decomposition of genetic variation revealed slightly higher levels of differentiation for females than for males (Table 1), this difference was not significant and pairwise F_ST_ values varied greatly (Table S3A and B). Examining genetic differentiation for a more locally restricted subset of chimpanzee social groups (Central Region, Figure 1), which was comparable to the bonobo sample, did not change the results qualitatively (Table 1). Including only groups in the AMOVA analysis that were sampled in both males and females yielded the same results (data not shown).

**Table 1:** Autosomal genetic differentiation (with 95% confidence interval) in bonobo and chimpanzee groups.

	Male			Female		
Species	Pairwise–comparisons	Autosomal F_ST_		Pairwise–comparisons	Autosomal F_ST_	
Bonobo	3	0,025	(0.004–0.050)	10	0,001	(0.000–0.016)
Western chimpanzee	21	0,021	(0.011–0.033)	28	0,016	(0.007–0.025)
Western chimpanzee Central region*	15	0,028	(0.016–0.042)	10	0,025	(0.014–0.037)

*We examined genetic differentiation for a more locally restricted subset of chimpanzee social groups (Central Region, Figure 1) which was comparable to the bonobo sample, and for the entire chimpanzee sample. Results, however, did not qualitatively change. To minimize stochasticity, for all analyses of genetic differentiation between communities we excluded social groups with fewer than four individuals genotyped at the respective marker (autosomal/Y-chromosomal). Therefore, the number of pairwise comparisons differs between the autosomal and Y-chromosomal data (Table 2).

**Table 2:** Unstandardized and standardized Y–chromosomal genetic differentiation (with 95% confidence interval) in bonobo and chimpanzee groups.

Species	Pairwise-comparisons	Y–F_ST_ unstandardized		Y–F_ST_ standardized	
Bonobo	6	0.915	(0.851–0.960)	0.964	(0.873–1.000)
Western chimpanzee	15	0.517	(0.368–0.601)	0.602	(0.414–0.729)
Western chimpanzee Central region*	10	0.562	(0.403–0.650)	0.657	(0.461–0.778)
Eastern chimpanzee**	6	0.830	(0.730–0.910)	0.970	(0.790–1.000)

*We examined genetic differentiation for a more locally restricted subset of chimpanzee social groups (Central Region, Figure 1) which was comparable to the bonobo sample, and for the entire chimpanzee sample. Results, however, did not qualitatively change. **Eastern chimpanzee data taken from Langergraber and colleagues [62]. To minimize stochasticity, for all analyses of genetic differentiation between communities we excluded social groups with fewer than four individuals genotyped at the respective marker (autosomal/Y-chromosomal). Therefore, the number of pairwise comparisons differs between the Y-chromosomal and autosomal data (Table 1).

A total of 18 Y–haplotypes were found in chimpanzees, while only seven Y–haplotypes were found in bonobos (Table S4). We found that 80% of chimpanzee and 50% of the bonobo groups had more than one Y–haplotype (N_Haplotype/group_ = 1–4), even though several groups had small sample sizes. Y–chromosomal variants did not reliably delineate social groups in either species and were shared not only between neighboring groups, but also over distances of ∼50 km in our chimpanzee sample (e.g. G2 and GTZ; Table S4). Most groups, however, were significantly differentiated from each other as revealed by F_ST_ analysis (Table S5A and B). Interestingly, AMOVA F_ST_ was significantly lower in western chimpanzees than bonobos, in both the geographically restricted and the full chimpanzee sample (Table 2). Standardizing calculations of genetic differentiation consistently increased F_ST_ values, especially in the chimpanzee sample, but did not qualitatively change the results (Table 2).

### Genetic variation within empirical and simulated social groups

**Figure 2:**
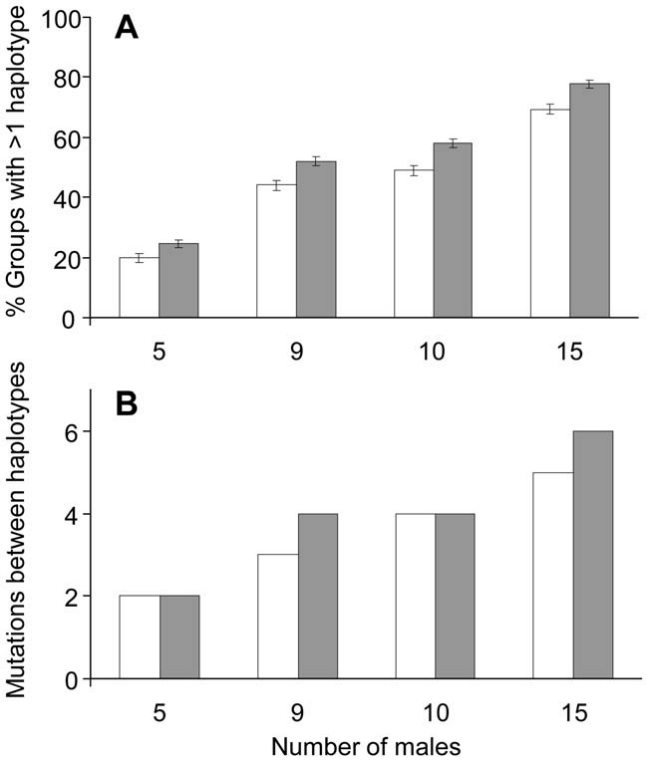
Simulations of Y–chromosomal variation in social groups of western chimpanzees and bonobos. Using empirically–based levels of reproductive skew, mutation rates and group sizes, we examined levels of haplotype diversity in terms of (A), number of haplotypes and (B), maximum number of mutational steps between Y–haplotypes that might arise within groups in the absence male–mediated gene flow. (A), the proportion of groups with more than one Y–haplotype and (B), the simulated maximum number of mutations possible between Y–haplotypes within groups increases with the number of males in the group. White bars indicate bonobos, grey bars indicate chimpanzees. Maximum numbers of mutations are only shown if observed in >1% of simulated groups. Average number of reproducing males in the habituated study groups  = 9. Error bars represent ± one standard deviation.

We first assessed whether it was plausible to find multiple Y–haplotypes in groups as often as was observed empirically (80% of western chimpanzee groups, 50% of bonobo groups) and over a sustained period in the absence of male–mediated gene flow. Using varied size groups of reproductively active males and realistic assumptions for the mutation rate and distribution of reproductive skew, we found that 20 – 78 and 17 – 69% of simulated chimpanzee and bonobo groups, respectively, contained multiple Y–haplotypes, overlapping with the empirical data in bonobos and approaching the levels observed in wild chimpanzee groups (Figure 2A). As expected from population genetics theory, where genetic drift decreases when group size increases, simulations showed that multiple Y–haplotypes were found more often in larger groups (Figure 2A).

We next examined how often highly divergent Y–haplotypes are likely to be observed within groups, and whether such divergent types are likely to be observed in the absence of male–mediated gene flow. We therefore determined the maximum number of mutational steps between Y–haplotypes arising within groups over time in the absence of gene flow, using the simulation conditions specified above. The simulations suggest that maximum numbers of mutations between Y–haplotypes observed within simulated groups at frequencies >1%, were 6 mutations in chimpanzees (frequency  = 1.3%, male group size = 15) and 5 mutations in bonobos (frequency  = 1.4 %, male group size = 15; Figure 2B). However, even more highly divergent Y–haplotypes were actually observed in one of the ten empirical chimpanzee groups (10 mutations) and two of the six bonobo groups (8 and 25 mutations).

Because this suggests that these highly divergent Y–haplotypes must stem from outside the social group, we then estimated rates of male–mediated gene flow compatible with the observed frequencies of such types. To create divergent Y–haplotypes within groups at a minimal frequency of 1/10 (10%) of groups in chimpanzees or 2/6 (33.3%) in bonobos, rates of male–mediated gene flow of 3.5 – 7% or 14.5 – 28.5% immigrant Y–haplotypes/generation, depending upon the group size, are necessary in chimpanzees and bonobos, respectively (Table 3).

**Table 3:** Estimated rates of male–mediated gene flow in bonobos and western chimpanzees for different male group sizes.

	Immigrant haplotypes/generation (%)	
N_males_	Bonobo	Western chimpanzee
5	28.5	7
9	19	4.5
10	17	4
15	14.5	3.5
Average	19.8	4.8

## Discussion

### Genetic differentiation between groups in chimpanzees and bonobos

We explored patterns of genetic differentiation and specifically the potential for male–mediated gene flow among social groups of western chimpanzees and bonobos. Autosomal genetic differentiation was similarly low in both species, and slightly but not significantly higher in males than in females. While this finding is consistent with *Pan* dispersal patterns [15], [16], it also suggests that nearly universal female dispersal is highly effective in hindering genetic differentiation among groups, and potentially that some degree of male–mediated gene flow also occurs. The lack of greater genetic differentiation at a larger scale, i.e. among pairs of groups separated by up to 100 km, in the western chimpanzees of the Taï National Park (Table 1, Table S3A) indicates that in the recent past no effective barriers to chimpanzee gene flow were present in that forest habitat. In the Taï chimpanzee population female dispersal seems not to be locally constrained by landscape features or preferences for familiar natal habitats, as suggested for mountain gorillas [24], [81], golden–brown mouse lemurs [82] and European grey wolves [83].

We found significantly less genetic differentiation at the Y–chromosome in western chimpanzees than in bonobos and eastern chimpanzees [Table 2; 62]. This suggests that substantial variation exists in the extent of male–transmitted genetic variation, and potentially male philopatry, among *Pan* populations. Variation in the effective extent of male philopatry has been suggested for other primates exhibiting some form of male philopatry, such as hamadryas baboons [13], woolly and spider monkeys [25] and patrilocal human societies [62].

### Evidence for male–mediated gene flow in Pan

Our observations of sharing of Y-chromosome haplotypes among nearby groups as well as those separated by ∼50 km, the lack of increased genetic differentiation at larger geographic scales (Table 2), and the high levels of Y-chromosome variation within groups are suggestive of either successful male–mediated gene flow between groups of western chimpanzees and bonobos and/or retention of ancestral variation within groups sharing common ancestors. In order to distinguish these scenarios more clearly, we turned to simulations of Y–chromosome variation in western chimpanzee and bonobo groups. Our simulations showed that while mutation alone is insufficient to generate multiple Y–haplotypes in very small groups, it suffices in groups averaging nine to 15 reproducing males. Yet, our analysis was highly conservative from the aspect of inferring male–mediated gene flow, because for simplicity we used non–overlapping generations in the simulations. By thus not accounting for the occurrence of related individuals in a group, we potentially overestimated the expected proportion of groups containing variation arising through mutation. Similarly, for simplicity our simulations used groups of a constant size, as assuming approximate constancy of group size is consistent with long term, large–scale inferences of expanding or constant effective population sizes of chimpanzee subspecies and bonobos [e.g. 84]. However, if groups fluctuate in size and sometimes contain very small numbers of males this would make it more difficult for variation to be maintained solely by mutation.

The simulations also showed that when using realistic mutation rates and reproductive skew parameters, social groups would not be expected to contain Y–haplotypes as divergent as those we observed in our empirical data. Thus, we estimated male–mediated gene flow to be, on average, 4.8% immigrant Y–haplotypes/generation in chimpanzees and 19.8% immigrant Y–haplotypes/generation in bonobos. This lower estimate of gene flow among western chimpanzees as compared to bonobos seems, at first look, inconsistent with lower overall levels of Y–chromosomal differentiation among this chimpanzee population, and several explanations are possible. However, both estimates are based on a small number of observations and are associated with a large degree of uncertainty, and the bonobo gene flow estimation in particular was based on fewer social groups, thus potentially harboring a larger stochastic component due to sampling variance. An alternative explanation would point to differences in male effective population sizes as contributing to differences in these estimates of Y-chromosomal genetic differentiation or male-mediated gene flow. However, male effective population size is not expected to differ greatly between western chimpanzees and bonobos as the long-term effective population sizes of both taxa are similar [85] and levels of short-term male reproductive skew are also similar [56].

Finally, we estimated gene flow by considering only highly dissimilar haplotypes as originating outside the group. It seems likely, however, that some of the highly similar haplotypes might also originate from outside the group. Our simulations showed that, particularly in smaller groups with 5 males, most often only one haplotype is present (as opposed to high variability within groups in the empirical sample) because mutation does not suffice to generate new variants frequently enough to counteract the loss of haplotypes when not all males get to reproduce. Thus, our estimated rate of male–mediated gene flow in the western chimpanzee population might be considered a minimum estimate and further study is needed in both western chimpanzees and bonobos.

### Potential mechanisms of male–mediated gene flow in Pan

Several mechanisms can facilitate male–mediated gene flow among groups. Despite years of cumulative study, quantitative data on population dynamic processes like group splits or takeovers that might redistribute genetic variation are limited in chimpanzees and bonobos [but see disintegration of chimpanzee groups and emigration of parous females; 37,46]. However, more information exists on individual behaviors that may allow male–mediated gene flow. Dispersal by adults of the more philopatric sex, as seen in many other mammals (banded mongoose [86]; Belding's ground squirrel [32]; bottlenose dolphin [87]; porcupine [88]; white–faced capuchin monkey [30]; woolly monkey [25]; but see [89]) appears to be possible in bonobos [44], while in chimpanzees intense and sometimes lethal aggression between males from different social groups would seem likely to prevent adult males from successfully integrating into new groups [37]–[41].

However, alternative mechanisms of male–mediated gene flow are possible. Female breeding dispersal with male offspring is rare in species such as *Pan* that practice mate– and resource–defense polygyny [5], but has been reported anecdotally from different chimpanzee groups [46]–[48] and also in bonobos (Hohmann, unpublished data). Alternatively, EGPs can form an important part of the mating system in many different birds and mammals (e.g. Alpine marmot [90]; fat–tailed dwarf lemur [91]; large tree shrew [92]; meerkats [93]), particularly in species where dominant males are not able to completely monopolize reproduction [35], [36], [94], [95]. In chimpanzees, extra–group conceptions occur, and observations suggest they may not solely relate to coercive mating by males from neighboring groups at times when females are solitary [41], but also that females may visit neighboring groups, sometimes over several weeks or months, and initiate copulations with males of the host group [37], [41], [48]. We suggest that EGPs are a likely mechanism for the inferred male–mediated gene flow in western chimpanzees. In the studied population a rate of extra group paternity of 6 – 10% has been inferred from genetic parentage analysis, and assuming half of the resulting offspring are male, this estimate is in good agreement with our inferred rate of male–mediated gene flow [54], [55]. In contrast to western chimpanzees, EGP has not been reported from other chimpanzee subspecies [57,58; K. Langergraber, unpublished data], suggesting that successful male–mediated gene flow through EGP could represent a true population or subspecies difference that shapes the genetic structure at male – transmitted markers differently in different populations.

Data from a small sample of offspring from one group of habituated bonobos suggested that 5% of male offspring were not sired by males within the group [56]. We are hesitant to conclude from our estimate of 19.8% male–mediated gene flow that EGP may not completely explain potential male–mediated gene flow in this species, especially in the light of relatively higher levels of Y–chromosomal genetic differentiation among groups and a presumably large sampling variance in the bonobo data due to the sampling of few social groups. Anecdotal behavioral evidence, such as the immigration of females with dependent male offspring and even adolescent or adult male dispersal [44], does however indicate that additional behavioral mechanisms might play a role in this species and deserve further investigation.

In sum, our results strongly suggest that male–mediated gene flow occurs at a detectable level in wild groups of two patrilocal species, western chimpanzees and bonobos, and is likely mediated behaviorally through male and female reproductive strategies. It also seems possible that successful male–mediated gene flow through EGP could represent an outcome of behavioral variants in chimpanzees that shape the genetic structure at male transmitted markers differently in different populations. Thus, as in humans [96], the extent to which male–mediated gene flow is limited by male philopatry seems to vary considerably among chimpanzee and bonobo populations. Our findings add to a growing body of evidence suggesting great behavioral diversity among the different species and subspecies of *Pan* [97]–[99].

## Supporting Information

Table S1 **Characteristics of 19 autosomal microsatellite markers used to genotype bonobos and western chimpanzees.** bp, base pairs; H_Obs_ , observed heterozygosity; H_Exp_, expected heterozygosity; N_alleles_, number of alleles.
(DOC)

Table S2 **Simulated distributions of reproductive skew among bonobo and chimpanzee males for groups of different sizes.** Values represent probabilities of transmission of an individual haplotype to the next generation and were derived from the original data using a best-fit log-function adjusted to the respective group sizes. Data were derived from human hunter-gatherer populations [79].
(DOC)

Table S3 **Pairwise autosomal genetic differentiation (F_ST_) in western chimpanzee (A) and bonobo (B) groups.** Comparisons among males are presented below the diagonal, and among females above the diagonal, with sample sizes in brackets. Significantly differentiated pairs (*p*<0.05) are shown in bold. To minimize stochasticity, for all analyses of genetic differentiation between communities we excluded social groups with fewer than four individuals genotyped at the respective marker (autosomal/Y-chromosomal). Therefore, the number of pairwise comparisons differs between the autosomal and Y-chromosomal data (Table S5). Bonobo group C3 was only genotyped at Y-chromosomal markers [61] and is therefore not included here.
(DOC)

Table S4 **Y-chromosomal microsatellite haplotypes of bonobo and western chimpanzee males.** Locality indicates sampling locations as shown in Figure 1. Individual Y-haplotypes at a locus are given as number of repeat units. Empty cells indicate missing values. Locus DYS588 and DYS562 did not amplify in bonobos, locus DYS632 was only typed in a few individuals, thus data for these loci are not used here. N, number of individuals sharing the Y-haplotype; n.a., not analyzed; ^a^, Y-chromosomal loci DYS; ^b^, Indel, coded as highest repeat number due to insufficient knowledge of mutational pattern.
(DOC)

Table S5 **Pairwise Y–chromosomal genetic differentiation (F_ST_) in western chimpanzee (A) and bonobo (B) groups.** Significantly differentiated pairs (*p*<0.05) are shown in bold. Sample sizes are indicated in brackets. To minimize stochasticity, for all analyses of genetic differentiation between communities we excluded social groups with fewer than four individuals genotyped at the respective marker (autosomal/Y-chromosomal). Therefore, the number of pairwise comparisons differs between the Y-chromosomal and autosomal data (Table S3).
(DOC)

Information S1 **Supplementary analytical procedures.** Two-step amplification of DNA from fecal samples.
(DOC)

Information S2 **Simulation source code.** The code is written in Java (Sun Microsystems Inc. 1994–2009).
(DOC)
