# Supplementary material for: Male-Mediated Gene Flow in Patrilocal Primates
Source: PLoS One. 2011 Jul 1;6(7):e21514. doi: 10.1371/journal.pone.0021514 (PMC3128582; doi:10.1371/journal.pone.0021514)
Supplement: Table S1 — Characteristics of 19 autosomal microsatellite markers used to genotype bonobos and western chimpanzees. bp, base pairs; HObs , observed heterozygosity; HExp, expected heterozygosity; Nalleles, number of alleles. (DOC) [file pone.0021514.s001.doc]

**Supplementary Table S1.**

|  | Western chimpanzee | | | | Bonobo | | | |
| --- | --- | --- | --- | --- | --- | --- | --- | --- |
| Locus | N_alleles_ | Allele size range (bp) | H_Obs_ | H_Exp_ | N_alleles_ | Allele size range (bp) | H_Obs_ | H_Exp_ |
| D1S1622 | 5 | 234–252 | 0.429 | 0.443 | 6 | 240–258 | 0.622 | 0.652 |
| D1S1656 | 10 | 110–150 | 0.726 | 0.682 | 8 | 126–154 | 0.783 | 0.796 |
| D2S1326 | 14 | 175–224 | 0.858 | 0.894 | 9 | 191–219 | 0.793 | 0.835 |
| D2S1329 | 10 | 153–205 | 0.832 | 0.805 | 4 | 173–185 | 0.705 | 0.695 |
| D3S2459 | 10 | 164–208 | 0.768 | 0.826 | 10 | 172–200 | 0.742 | 0.709 |
| D3S3038 | 10 | 162–198 | 0.814 | 0.793 | 7 | 178–198 | 0.686 | 0.690 |
| D4S1627 | 7 | 201–233 | 0.816 | 0.748 | 6 | 189–221 | 0.733 | 0.726 |
| D5S1457 | 7 | 94–118 | 0.762 | 0.774 | 10 | 102–134 | 0.884 | 0.876 |
| D5S1470 | 8 | 169–197 | 0.838 | 0.810 | 11 | 197–237 | 0.840 | 0.826 |
| D6S1056 | 8 | 222–250 | 0.693 | 0.714 | 6 | 226–246 | 0.756 | 0.760 |
| D7S817 | 12 | 115–159 | 0.894 | 0.872 | 7 | 106–131 | 0.747 | 0.785 |
| D7S2204 | 12 | 149–197 | 0.805 | 0.798 | 6 | 141–161 | 0.884 | 0.773 |
| D9S910 | 7 | 97–118 | 0.816 | 0.782 | 7 | 100–118 | 0.783 | 0.790 |
| D10S676 | 7 | 149–173 | 0.740 | 0.729 | 6 | 141–169 | 0.753 | 0.796 |
| D11S2002 | 9 | 137–173 | 0.866 | 0.835 | 11 | 129–177 | 0.824 | 0.812 |
| D12S66 | 10 | 141–178 | 0.732 | 0.754 | 6 | 129–157 | 0.750 | 0.700 |
| D14S30 | 11 | 185–225 | 0.850 | 0.833 | 9 | 218–250 | 0.775 | 0.775 |
| D16S2624 | 5 | 110–126 | 0.702 | 0.716 | 4 | 110–126 | 0.722 | 0.600 |
| D18S536 | 6 | 143–163 | 0.553 | 0.512 | 5 | 135–151 | 0.646 | 0.624 |
| Mean | 8.8 |  | 0.76 | 0.75 | 7.3 |  | 0.76 | 0.75 |
