# Supplementary material for: Male-Mediated Gene Flow in Patrilocal Primates
Source: PLoS One. 2011 Jul 1;6(7):e21514. doi: 10.1371/journal.pone.0021514 (PMC3128582; doi:10.1371/journal.pone.0021514)
Supplement: Table S2 — Simulated distributions of reproductive skew among bonobo and chimpanzee males for groups of different sizes. Values represent probabilities of transmission of an individual haplotype to the next generation and were derived from the original data using a best-fit log-function adjusted to the respective group sizes. Data were derived from human hunter-gatherer populations [79]. (DOC) [file pone.0021514.s002.doc]

**Supplementary Table S2.**

| N_males_ | Skew distribution |  |
| --- | --- | --- |
| 5 | 0.37, 0.25, 0.17, 0.12, 0.09 | |
| 9 | 0.24, 0.18, 0.14, 0.11, 0.09, 0.08, 0.06, 0.05, 0.05 | |
| 10 | 0.22, 0.17, 0.13, 0.11, 0.09, 0.08, 0.07, 0.05, 0.05, 0.03 | |
| 15 | 0.16, 0.13, 0.11, 0.09, 0.08, 0.07, 0.06, 0.06, 0.05, 0.04, 0.04, 0.03, 0.03, 0.03, 0.02 | |
