# Supplementary material for: Male-Mediated Gene Flow in Patrilocal Primates
Source: PLoS One. 2011 Jul 1;6(7):e21514. doi: 10.1371/journal.pone.0021514 (PMC3128582; doi:10.1371/journal.pone.0021514)
Supplement: Table S3 — Pairwise autosomal genetic differentiation (FST) in western chimpanzee (A) and bonobo (B) groups. Comparisons among males are presented below the diagonal, and among females above the diagonal, with sample sizes in brackets. Significantly differentiated pairs (p<0.05) are shown in bold. To minimize stochasticity, for all analyses of genetic differentiation between communities we excluded social groups with fewer than four individuals genotyped at the respective marker (autosomal/Y-chromosomal). Therefore, the number of pairwise comparisons differs between the autosomal and Y-chromosomal data (Table S5). Bonobo group C3 was only genotyped at Y-chromosomal markers [61] and is therefore not included here. (DOC) [file pone.0021514.s003.doc]

**Supplementary Table S3.**

| **A** | East (8) | G2 (10) | G4 (4) | GTZ | Meteo (13) | Middle (7) | N2 (6) | North (14) | South (31) |
| --- | --- | --- | --- | --- | --- | --- | --- | --- | --- |
| East (10) |  | 0.004 | 0.000 | n.a. | 0.016 | 0.020 | 0.000 | **0.025** | 0.003 |
| G2 (7) | 0.000 |  | 0.002 | n.a. | 0.010 | 0.014 | 0.000 | **0.018** | **0.014** |
| G4 | n.a. | n.a. |  | n.a. | 0.000 | 0.011 | 0.000 | **0.030** | 0.000 |
| GTZ (8) | **0.031** | 0.001 | n.a. |  | n.a. | n.a. | n.a. | n.a. | n.a. |
| Meteo (6) | 0.015 | 0.000 | n.a. | 0.000 |  | 0.010 | 0.000 | **0.036** | 0.006 |
| Middle (4) | **0.047** | 0.011 | n.a. | **0.039** | 0.049 |  | 0.000 | **0.043** | **0.026** |
| N2 | n.a. | n.a. | n.a. | n.a. | n.a. | n.a. |  | **0.023** | 0.000 |
| North (9) | 0.011 | 0.000 | n.a. | **0.027** | 0.014 | 0.026 | n.a. |  | **0.044** |
| South (26) | **0.019** | 0.000 | n.a. | **0.019** | 0.000 | **0.037** | n.a. | **0.024** |  |

| **B** | C2 (14) | C1 (17) | C4 (12) | C6 (8) | C5 (4) |
| --- | --- | --- | --- | --- | --- |
| C2 (10) |  | 0.000 | 0.000 | **0.017** | 0.000 |
| C1 (11) | **0.032** |  | 0.000 | **0.026** | 0.000 |
| C4 (9) | **0.020** | **0.031** |  | 0.011 | 0.000 |
| C6 | n.a. | n.a. | n.a. |  | 0.029 |
| C5 | n.a. | n.a. | n.a. | n.a. |  |
