# Supplementary material for: Male-Mediated Gene Flow in Patrilocal Primates
Source: PLoS One. 2011 Jul 1;6(7):e21514. doi: 10.1371/journal.pone.0021514 (PMC3128582; doi:10.1371/journal.pone.0021514)
Supplement: Table S4 — Y-chromosomal microsatellite haplotypes of bonobo and western chimpanzee males. Locality indicates sampling locations as shown in Figure 1. Individual Y-haplotypes at a locus are given as number of repeat units. Empty cells indicate missing values. Locus DYS588 and DYS562 did not amplify in bonobos, locus DYS632 was only typed in a few individuals, thus data for these loci are not used here. N, number of individuals sharing the Y-haplotype; n.a., not analyzed; a, Y-chromosomal loci DYS; b, Indel, coded as highest repeat number due to insufficient knowledge of mutational pattern. (DOC) [file pone.0021514.s004.doc]

**Supplementary Table S4.**

| Western chimpanzee | | | | | | | | | | | | | | | |
| --- | --- | --- | --- | --- | --- | --- | --- | --- | --- | --- | --- | --- | --- | --- | --- |
| Locality | Haplotype | N | Y392^a^ | Y439 | Y469 | Y502 | Y510 | Y517 | Y520 | Y533 | Y612 | Y630 | Y562 | Y588 | Y632 |
| East | WE01 | 3 | 1 | 1 | 1 | 2 | 2 | 4 | 3 | 2 | 4 | 7 | 2 | 3 | 1 |
|  | WE02 | 2 | 3 | 1 | 1 | 1 | 3 | 1 | 3 | 2 | 4 | 7 | 4 | 3 | 1 |
|  | WE03 | 2 | 3 | 1 | 1 | 1 | 3 | 1 | 3 | 2 | 3 | 7 | 3 | 2 | 1 |
|  | WE04 | 1 | 3 | 1 | 1 | 1 | 3 | 1 | 3 | 2 | 4 | 7 | 4 | 2 | 1 |
| G2 | WS03 | 2 | 3 | 1 | 1 | 1 | 3 | 1 | 3 | 2 | 4 | 7 | 3 | 2 | 1 |
|  | WGTZ01 | 3 | 3 | 1 | 1 | 1 | 2 | 1 | 3 | 2 | 4 | 7 | 3 | 2 | 1 |
|  | WMet01 | 1 | 3 | 1 | 1 | 1 | 3 | 1 | 3 | 2 | 5 | 7 | 3 | 2 | 1 |
|  | WG201 | 1 | 3 | 1 | 1 | 1 | 2 | 1 | 3 | 2 | 4 | 8 | 3 | 2 | 1 |
| G4 | WS03 | 2 | 3 | 1 | 1 | 1 | 3 | 1 | 3 | 2 | 4 | 7 | 3 | 2 | 1 |
|  | WG401 | 1 | 3 | 1 | 1 | 1 | 4 | 1 | 3 | 2 | 4 | 7 | 3 | 2 | 1 |
| GTZ | WGTZ01 | 4 | 3 | 1 | 1 | 1 | 2 | 1 | 3 | 2 | 4 | 7 | 3 | 2 | 1 |
|  | WGTZ02 | 2 | 3 | 1 | 1 | 1 | 2 | 1 | 3 | 2 | 4 | 6 | 3 | 2 | 1 |
| Meteo | WS03 | 1 | 3 | 1 | 1 | 1 | 3 | 1 | 3 | 2 | 4 | 7 | 3 | 2 | 1 |
|  | WGTZ01 | 1 | 3 | 1 | 1 | 1 | 2 | 1 | 3 | 2 | 4 | 7 | 3 | 2 | 1 |
|  | WMet01 | 1 | 3 | 1 | 1 | 1 | 3 | 1 | 3 | 2 | 5 | 7 | 3 | 2 | 1 |
| Middle | WS03 | 4 | 3 | 1 | 1 | 1 | 3 | 1 | 3 | 2 | 4 | 7 | 3 | 2 | 1 |
| N1 | WN101 | 1 | 1 | 4 | 1 | 2 | 3 | 4 | 3 | 2 | 4 | 1 | 2 | 4 | 1 |
|  | WN102 | 1 | 1 | 4 | 1 | 2 |  | 4 | 3 | 2 | 4 | 2 |  | 4 | 1 |
| N2 | WN201 | 2 | 3 | 4 | 1 | 2 | 3 | 5 | 4 | 1 | 6 | 3 | 1 | 3 | 1 |
| North | WN01 | 3 | 3 | 2 | 1 | 1 | 3 | 1 | 3 | 2 | 5 | 7 | 3 | 2 | 1 |
|  | WN02 | 1 | 3 | 2 | 1 | 1 | 3 | 1 | 3 | 2 | 5 | 8 | 3 | 2 | 1 |
| South | WS01 | 11 | 3 | 1 | 1 | 1 | 2 | 1 | 3 | 2 | 3 | 7 | 3 | 2 | 1 |
|  | WS02 | 2 | 3 | 2 | 1 | 1 | 2 | 1 | 3 | 2 | 3 | 7 | 3 | 2 | 1 |
|  | WS03 | 1 | 3 | 1 | 1 | 1 | 3 | 1 | 3 | 2 | 4 | 7 | 3 | 2 | 1 |
|  | WS04 | 1 | 3 | 2 | 1 | 1 | 2 | 1 | 3 | 2 | 4 | 7 | 3 | 2 | 1 |
| Bonobo | | | | | | | | | | | | | | | |
| Locality | Haplotype | N | Y392 | Y439 | Y469 | Y502 | Y510 | Y517 | Y520 | Y533 | Y612 | Y630 | Y562 | Y588 | Y632 |
| C1 | BSa03 | 10 | 6 | 4 | 5 | 1 | 1 | 3 | 2 | 1 | 1 | 4 | n.a. | n.a. | n.a. |
| C2 | BSa01 | 12 | 1 | 4 | 1 | 5 | 1 | 1 | 2 | 3 | 6 | 3 | n.a. | n.a. | n.a. |
|  | BSa02 | 3 | 1 | 4 | 1 | 5 | 1 | 1 | 2 | 3 | 6 | 7^b^ | n.a. | n.a. | n.a. |
| C3 | BSa01 | 1 | 1 | 4 | 1 | 5 | 1 | 1 | 2 | 3 | 6 | 3 | n.a. | n.a. | n.a. |
|  | BSa06 | 3 | 1 | 3 | 1 | 4 | 2 | 3 | 4 | 4 | 6 | 3 | n.a. | n.a. | n.a. |
|  | BSa07 | 1 | 1 | 3 | 1 | 4 | 1 | 3 | 4 | 4 | 6 | 3 | n.a. | n.a. | n.a. |
|  | BSa08 | 1 | 1 | 3 | 1 | 4 | 2 | 3 | 2 | 4 | 6 | 3 | n.a. | n.a. | n.a. |
| C4 | BSa03 | 6 | 6 | 4 | 5 | 1 | 1 | 3 | 2 | 1 | 1 | 4 | n.a. | n.a. | n.a. |
| C5 | BSa03 | 2 | 6 | 4 | 5 | 1 | 1 | 3 | 2 | 1 | 1 | 4 | n.a. | n.a. | n.a. |
| C6 | BSa03 | 2 | 6 | 4 | 5 |  | 1 | 3 | 2 | 1 | 1 | 4 | n.a. | n.a. | n.a. |
|  | BSa05 | 1 | 1 | 1 | 1 | 4 | 2 | 1 | 2 | 3 | 6 | 7 | n.a. | n.a. | n.a. |
