# Supplementary material for: Male-Mediated Gene Flow in Patrilocal Primates
Source: PLoS One. 2011 Jul 1;6(7):e21514. doi: 10.1371/journal.pone.0021514 (PMC3128582; doi:10.1371/journal.pone.0021514)
Supplement: Table S5 — Pairwise Y–chromosomal genetic differentiation (FST) in western chimpanzee (A) and bonobo (B) groups. Significantly differentiated pairs (p<0.05) are shown in bold. Sample sizes are indicated in brackets. To minimize stochasticity, for all analyses of genetic differentiation between communities we excluded social groups with fewer than four individuals genotyped at the respective marker (autosomal/Y-chromosomal). Therefore, the number of pairwise comparisons differs between the Y-chromosomal and autosomal data (Table S3). (DOC) [file pone.0021514.s005.doc]

**Supplementary Table S5.**

| **A** | East | G2 | GTZ | Middle | North | South |
| --- | --- | --- | --- | --- | --- | --- |
| East (8) |  |  |  |  |  |  |
| G2 (7) | **0.276** |  |  |  |  |  |
| GTZ (6) | **0.368** | 0.151 |  |  |  |  |
| Middle (4) | **0.256** | 0.222 | **0.758** |  |  |  |
| North (4) | **0.488** | **0.671** | **0.851** | **0.889** |  |  |
| South (15) | **0.504** | **0.469** | **0.551** | **0.725** | **0.774** |  |

| **B** | C2 | C1 | C4 | C3 |
| --- | --- | --- | --- | --- |
| C2 (15) |  |  |  |  |
| C1 (10) | **0.970** |  |  |  |
| C4 (6) | **0.964** | 0.000 |  |  |
| C3 (6) | **0.803** | **0.894** | **0.856** |  |
