# Supplementary material for: Male-Mediated Gene Flow in Patrilocal Primates
Source: PLoS One. 2011 Jul 1;6(7):e21514. doi: 10.1371/journal.pone.0021514 (PMC3128582; doi:10.1371/journal.pone.0021514)
Supplement: Information S1 — Supplementary analytical procedures. Two-step amplification of DNA from fecal samples. (DOC) [file pone.0021514.s006.doc]

**Supplementary Information 1.**

We genotyped DNA extracts at 19 autosomal and 13 (chimpanzee) or 10 (bonobo) Y–chromosomal loci using a two–step amplification method as described in [1]. In an initial multiplexing step, all autosomal or Y-chromosomal microsatellite loci were amplified in a single reaction in 20-μL reaction volumes consisting of 1× SuperTaq buffer (HT Biotechnology), 1.75 mm MgCl2, 0.15 mm of each forward (unlabelled) and reverse (unnested) primer, 110 μm of each dNTP, 16 μg bovine serum albumin (BSA), 0.5 U SuperTaq (HT Biotechnology) premixed 2:1 with TaqStart Antibody (BD Biosciences), and 5 μL template DNA. PCR thermocycling was performed in a PTC-200 thermocycler (MJ Research) with the following parameters: initial denaturation for 9 min at 94 °C, 30 cycles of 20 s at 94 °C, 30 s at 55 °C and 30 s at 72 °C, and a final extension of 4 min at 72 °C. Initially, three to four independent amplifications of each sample were performed in 96-well plates (depending on the DNA quantity in the sample [1]), along with a minimum of five negative controls (where 5 μL H2O rather than DNA was added to the well).

Singleplex PCRs were carried out as above but with the following modifications: 5 μL of 1:100 diluted multiplex PCR product was used as template, half the amount of MgCl2 (0.875 mm) was added, and only 0.35 U of SuperTaq premixed 2:1 with TaqStart antibody was used. Furthermore, each singleplex PCR contained a single primer pair: 0.25 mm of a FAM, HEX or NED fluorescently

labelled forward primer and 0.25 mm of a reverse primer, which was nested in some cases. Finally, the cycling conditions were as above except primer-specific annealing

temperatures were used for each singleplex PCR (see [1] for autosomal loci; see [2] for Y-chromosomal loci). To guard against contamination, all steps of the PCR set-up (except the addition of template) were performed under a hood that was ultraviolet (UV) irradiated before and after use.

Up to four different PCR products from the second singleplex amplification step were combined and electrophoresed on an ABI PRISM 3100 Genetic Analyser and alleles were sized relative to an internal size standard (ROX labelled HD400) using GeneMapper Software version 3.7 (Applied Biosystems).

**Supplementary References**

1. Arandjelovic M, Guschanski K, Schubert G, Harris TR, Thalmann O, et al. (2009) Two-step multiplex polymerase chain reaction improves the speed and accuracy of genotyping using DNA from noninvasive and museum samples. Molecular Ecology Resources 9: 28-36.

2. Arandjelovic M, Head J, Rabanal LI, Schubert G, Mettke E, et al. (2011) Non-invasive genetic monitoring of wild central chimpanzees. PLoS ONE 6: e14761.
