# Supplementary material for: Male-Mediated Gene Flow in Patrilocal Primates
Source: PLoS One. 2011 Jul 1;6(7):e21514. doi: 10.1371/journal.pone.0021514 (PMC3128582; doi:10.1371/journal.pone.0021514)
Supplement: Information S2 — Simulation source code. The code is written in Java (Sun Microsystems Inc. 1994–2009). (DOC) [file pone.0021514.s007.doc]

**Supplementary Information 2.**

import java.util.ArrayList;

import java.util.Arrays;

/**

* This takes parameters (mutation rate, migration etc.) and returns the outcome (haplotype distributions in groups)

*/

public class Simulation{

public int groupSize = 10;

//the size of the group

public double mutationRate = 0.01;

//the mutation rate (range 1-0)

//this is the chance of a given individual for mutating in a given generation

//mutations occur only once and they occur before offspring are generated

public double migrationRate = 0.01;

//the migration rate (range 1-0)

//this is the probability of a single migrant appearing in a given generation

//(technically this should be a distribution, probably Poisson)

public double[] reproductiveSkew = {0.400,0.240,0.144,0.0864,0.0518};

//the percentage of all offspring a male of a given rank will sire

//for instance, the alpha sires 40% and the next highest 24%

public int groups = 100;

//the number of groups to simulate

public int generations = 100;

//the number of generations a simulation should take

public static final int FIXATED = 0;

//group state in which all haplotypes are identical

public static final int SIMILAR = 1;

//group state in which all haplotypes are closely related or identical

public static final int DISSIMILAR = 2;

//group state in which some haplotypes are not closely related

public int startingState = DISSIMILAR;

public static final int DISTANCE_OF_DIVERGENT_HAPLOTYPES = 10;

//the distance (in mutational steps) between haplotypes that are considered divergent

class Haplotype{

final Haplotype ancestor;

final double[] ID;

//randomly generated ID (big enough to be unique)

//this is used to distinguish different haplotypes

public Haplotype(Haplotype ancestor){

this.ID = getGUID();

this.ancestor = ancestor;

}

/**

* Returns the last recorded ancestor. This is equal to the haplotype for original haplotypes and migrants.

* For mutants, this is equal to a previous haplotype.

* @return

*/

public Haplotype getOrigin(){

Haplotype h = this;

while(h.ancestor!=null){

h = h.ancestor;

}

return h;

}

/**

* Returns the most recent ancestor. For original haplotypes and migrants, this is equal to the haplotype

* @return

*/

public Haplotype getImmediateAncestor(){

return (ancestor==null)? this:ancestor;

}

/**

* Generates a new haplotype via mutation from this.

*/

public Haplotype getMutant(){

return new Haplotype(this);

}

public boolean equals(Haplotype h){

return ID[0] == h.ID[0] && ID[1] == h.ID[1];

}

/**

* this is used for debugging only - returns an easy - to - read string

*/

public String toString(){

return "H "+(int)(ID[0]*1000)+"/"+(int)(ID[1]*1000) + ((ancestor!=null)? " - "+ancestor.toString():"");

}

}

class Individual{

private final Haplotype haplotype;

public Individual(Haplotype h){

this.haplotype = h;

}

public Haplotype getHaplotype(){

return haplotype;

}

public String toString(){

return haplotype.toString();

}

}

/**

* @return a Globally Unique IDentifier

* (a randomly generated value from such a large set that the probability of duplication is infinitesimal)

*/

public double[] getGUID(){

double [] GUID = {Math.random(), Math.random()};

return GUID;

}

ArrayList<Haplotype> haplotypes = null;

//store all haplotypes, ignoring how many individuals each one has

//ArrayList<Individual> individualsOfRank = null;

//keep separate pointers to all individuals of rank

ArrayList<Individual> individuals = null;

//all individuals

//this list represents a given generation

private void createStartingGeneration(){

haplotypes = new ArrayList<Haplotype>();

individuals = new ArrayList<Individual>();

if(startingState == DISSIMILAR){

for(int i=0;i<groupSize;i++){

Haplotype h = new Haplotype(null);

//create a new haplotype with no known ancestor

individuals.add(new Individual(h));

haplotypes.add(h);

}

}

else if(startingState == FIXATED){

Haplotype h = new Haplotype(null);

haplotypes.add(h);

for(int i=0;i<groupSize;i++){

individuals.add(new Individual(h));

}

}

}

private void newGeneration(){

//mutate individuals

for(int i=0;i<individuals.size();i++){

if(Math.random()<mutationRate){

Haplotype h = individuals.get(i).getHaplotype();

Haplotype mutant = h.getMutant();

haplotypes.add(mutant);

individuals.set(i, new Individual(mutant));

}

}

//add a migrant

if(Math.random()<migrationRate){

Haplotype migrantHaplotype = new Haplotype(null);

haplotypes.add(migrantHaplotype);

individuals.add(new Individual(migrantHaplotype));

}

//set the ranks in the group

setRanks();

//generate offspring

ArrayList<Individual> offspring = new ArrayList<Individual>();

double sum = 1;

int n = Math.min(individuals.size(), reproductiveSkew.length);

//only use the first n values in case group has fewer members

//than skew is defined for

for(int i=0;i<n;i++){

sum -= reproductiveSkew[i];

}

//let the size of the new group not be equal to the size of the old one,

//but to the fixed group size given at the beginning

//formula that gives each male's chance of fathering a given individual:

//P(x) = reproductiveSkew(x) + (1 - sum(reproductiveSkew))/individuals

outer: for(int i=0;i<groupSize;i++){

//number of new individuals = fixed group size, not previous group size!

double probability = 0;

double random = Math.random();

for(int j=0;j<individuals.size();j++){

probability += sum/((double)individuals.size());

if(j<reproductiveSkew.length){

probability += reproductiveSkew[j];

}

if(random<=probability){

offspring.add(new Individual(individuals.get(j).getHaplotype()));

continue outer;

}

}

}

//set the current generation to be the new one

individuals = offspring;

}

/**

* Set the ranks in the group.

* Make the first n individuals in the list have the first n positions in rank(e.g. the alpha is at index 0)

*/

private void setRanks(){

ArrayList<Individual> individualsOfRank = new ArrayList<Individual>();

for(int i=0; i< Math.min(reproductiveSkew.length, individuals.size());i++){

int index = (int)Math.floor(Math.random()*individuals.size());

if(index==individuals.size()){

index--;

}

individualsOfRank.add(individuals.get(index));

individuals.remove(index);

}

individualsOfRank.addAll(individuals);

individuals.clear();

individuals.addAll(individualsOfRank);

}

//=============================================================================================================================

/*These methods are used to run the simulation for specific analyses

*/

public double[][] runForDivergence(){

double[][] data = new double[generations][3];

//holds data: divergent - similar - fixed

for(int i=0;i<generations;i++){

Arrays.fill(data[i], 0);

}

for(int i=0;i<groups;i++){

createStartingGeneration();

for(int j=0;j<generations;j++){

newGeneration();

double distance = getGreatestDistanceBetweenHaplotypes();

if(distance==0){

//fixed

data[j][2]++;

}

else if(distance < DISTANCE_OF_DIVERGENT_HAPLOTYPES){

//similar

data[j][1]++;

}

else data[j][0]++;//divergent

}

}

return data;

}

//the generation given as output is the number of generations the haplotype persists,

//which is equal to the number of new generations until it disappears

//e.g. a haplotype that is present in the starting generation but disappears after the first generation is considered

//to have persisted for one generation

public int[] runForHaplotypePersistence(){

int[] data = new int[generations+1];

Arrays.fill(data,0);

for(int i=0;i<groups;i++){

createStartingGeneration();

Haplotype founder = individuals.get(0).getHaplotype();

boolean persist = true;

for(int j=0;j<generations;j++){

newGeneration();

if(!isHaplotypePresent(founder)){

data[j+1]++;

//add one to the data array at a slot specified by the current generation

persist = false;

break;

}

}

if(persist){

data[0]++;

}

}

return data;

}

public double[][] runForHaplotypeDistance(){

double[][] data = new double[generations][groups];

for(int i=0;i<groups;i++){

createStartingGeneration();

for(int j=0;j<generations;j++){

newGeneration();

data[j][i] = getGreatestDistanceBetweenHaplotypes();

}

}

return data;

}

//=============================================================================================================================

/**

* The following methods are used while running a simulation to analyze the results.

*/

private boolean isHaplotypePresent(Haplotype haplotype){

for(int i=0;i<individuals.size();i++){

if(individuals.get(i).getHaplotype().equals(haplotype)){

return true;

}

}

return false;

}

//This returns the greatest distance, in mutational steps, between two haplotypes in a group.

//Haplotypes that are not related via mutation (because they are starting haplotypes in a group with all - different haplotypes or because

//one arose via migration) have infinite distance

public double getGreatestDistanceBetweenHaplotypes(){

double distance = -1;

for(int i=0;i<individuals.size();i++){

for(int j=i+1;j<individuals.size();j++){

double dist = getDistanceBetweenHaplotypes(individuals.get(i).getHaplotype(), individuals.get(j).getHaplotype());

if(distance<dist)distance=dist;

}

}

return distance;

}

//get the mutational steps separating two haplotypes

//algorithm:

//loop:

//go through the ancestors of h2 and if h1 is found, return the number of steps needed + the number of steps h1 was shifted back

//set h1 to be its ancestor (shift it back)

private double getDistanceBetweenHaplotypes(Haplotype h1, Haplotype h2){

int steps = 0;

while(true){

double d = getDistanceAlongBranch(h2, h1);

if(d!=Double.POSITIVE_INFINITY){

return d+steps;

}

if(h1 == h1.getImmediateAncestor()){

return Double.POSITIVE_INFINITY;

}

h1 = h1.getImmediateAncestor();

steps++;

}

}

//return the distance between two haplotypes on a graph in terms of mutation steps

//assuming that one is an ancestor of the other

//used by getDistanceBetweenHaplotypes(h1,h2)

private double getDistanceAlongBranch(Haplotype h, Haplotype ancestor){

int distance = 0;

while(true){

if(h.equals(ancestor)){

return distance;

}

else if(h==h.getImmediateAncestor()){

break;

}

h = h.getImmediateAncestor();

distance++;

}

return Double.POSITIVE_INFINITY;

}

/**

* The following methods are only used by OPSim (program that estimates most likely

* parameters based on outcome)

*/

public double[][] run(){

double[][] data = new double[generations][5];

for(int i=0;i<generations;i++){

Arrays.fill(data[i], 0);

}

//initialize array to 0

//so values can be added

for(int i=0;i<groups;i++){

int[][] singleData = runSingleSimulation();

//structure of singleData array:

//{group state as number in range 0-2, migration metric, mutation metric}

for(int j=0;j<generations;j++){

data[j][singleData[j][0]]++;

//add 1 to a certain field of the three fields at the start

//of the array, as determined by the group state

data[j][3]+=singleData[j][1];

data[j][4]+=singleData[j][2];

//add the migration metric and the mutation metric to the 4th and 5th fields of the array

}

}

for(int i=0;i<data.length;i++){

data[i][3]/=(double)(groups);

data[i][4]/=(double)(groups);

//divide metrics by the number of groups simulated

//to obtain an average

}

return data;

}

private int[][] runSingleSimulation(){

int[][] data = new int[generations][3];

createStartingGeneration();

//note that the data of the starting generation are not saved

for(int i=0;i<generations;i++){

newGeneration();

data[i][0] = getGroupState();

int[] metrics = getGroupMetrics();

if(metrics[0]!=0 || metrics[1]!=0){

int d = 4;

}

//add the metrics to the end of the array

data[i][1] = metrics[0];

data[i][2] = metrics[1];

}

return data;

}

/**

* Return numbers quantifying the effects of migration and mutation. The generation can be seen as a graph, with each haplotype forming a vertex and

* related haplotypes connected by edges. The migration metric is then equal to the number of connected components minus one. The mutation metric is equal to the number of vertexes in each connected component minus the number of connected components,

* i.e. sum(vertexes -1 for each connected component)

* @return

*/

private int[] getGroupMetrics(){

ArrayList<ArrayList<Haplotype>> connectedComponents = new ArrayList<ArrayList<Haplotype>>();

outer: for(int i=0;i<individuals.size();i++){

Haplotype h1 = individuals.get(i).getHaplotype();

middle: for(int j=0; j<connectedComponents.size();j++){

ArrayList<Haplotype> connectedComponent = connectedComponents.get(j);

inner: for(int k=0;k<connectedComponent.size();k++){

Haplotype h2 = connectedComponent.get(k);

if(h1.equals(h2)){

//if the two haplotypes are identical, do not add the new haplotype to any connected component

continue outer;

}

if(!(h1.getOrigin().equals(h2.getOrigin()))){

//if the two haplotypes are not related, then try to add the new haplotype to a subsequent connected component

continue middle;

}

}

//if this loop has been passed, then the haplotype must be related to each haplotype in the connected component

//but not identical to any of them

connectedComponent.add(h1);

continue outer;

}

//if this loop has been passed, then the haplotype must not be related to any haplotype already examined

//so it should form its own component

ArrayList<Haplotype> component = new ArrayList<Haplotype>();

component.add(h1);

connectedComponents.add(component);

}

int migrationMetric = connectedComponents.size() - 1;

int mutationMetric = 0;

for(ArrayList connectedComponent: connectedComponents){

//mutationMetric += (connectedComponent.size() - 1); - old approach, no mutational distance

mutationMetric += getMutationalSteps(connectedComponent);

}

int[] result = {migrationMetric, mutationMetric};

return result;

}

/**

* Return the state of the group (whether all haplotypes are the same, whether some are similar or whether all are different)

* @param haplotypes

* @return

*/

private int getGroupState(){

if(individuals.size()<1){

return -1;

}

//method: go through list of individuals once

//compare the first haplotype with every other haplotype

//this is enough to be able to take this measure of the group

Haplotype h1 = individuals.get(0).getHaplotype();

Haplotype h1_origin = h1.getOrigin();

int state = FIXATED;

for(int i=1;i<individuals.size();i++){

Haplotype h2 = individuals.get(i).getHaplotype();

if(h1.equals(h2)){

continue;

}

Haplotype h2_origin = h2.getOrigin();

if(h1_origin.equals(h2_origin)){

if(state!=DISSIMILAR){

state = SIMILAR;

}

}

else {

state = DISSIMILAR;

//one dissimilar pair makes the entire state dissimilar

break;

}

}

return state;

}

private int getMutationalSteps(ArrayList<Haplotype> haplotypes){

ArrayList<Haplotype> visitedHaplotypes = new ArrayList<Haplotype>();

//first get original haplotype

//there can be only one such haplotype

Haplotype original = null;

for(int i=0;i<haplotypes.size();i++){

Haplotype h = haplotypes.get(i);

if(h.equals(h.getOrigin())){

original = h;

break;

}

}

//add the original haplotype to the list of visited haplotypes

visitedHaplotypes.add(original);

int distance = 0;

for(int i=0;i<haplotypes.size();i++){

Haplotype h = haplotypes.get(i);

while(h!=null){

if(visitedHaplotypes.contains(h)){

break;

}

distance++;

visitedHaplotypes.add(h);

h = h.getImmediateAncestor();

}

}

return distance;

}

public String getSimulationNameSuffix(){

return "mut="+mutationRate+", "+"mig="+migrationRate+", "+"size="+groupSize+", "+"groups="+groups+", "+"gen="+generations;

}

/**

* The following methods return text describing the results of the simulation. There is one method for each type of simulation

* that can be run, plus getParameters() which gives the starting parameters.

* The text is formatted so it can be saved as a csv file that microsoft excel will recognize.

*/

public String getParameters(){

StringBuilder sb = new StringBuilder();

String N = System.getProperty("line.separator");

String D = ";";//this is the default delimiter for excel

sb.append("Chimpanzee group structure simulation"+N);

sb.append(N);

sb.append("mutation rate:"+D+format(mutationRate)+N);

sb.append("migration rate:"+D+format(migrationRate)+N);

sb.append("group size:"+D+groupSize+N);

sb.append("number of groups:"+D+groups+N);

sb.append("number of generations:"+D+generations+N);

sb.append("starting group state:"+D+((startingState==DISSIMILAR)? "all different haplotypes":"all identical haplotypes")+N);

sb.append(N);

sb.append("reproductive skew:"+N);

for(int i=0;i<reproductiveSkew.length;i++){

sb.append(format(reproductiveSkew[i])+D);

}

sb.append(N+N);

return sb.toString();

}

public String getDescriptionForDivergence(double[][] data){

StringBuilder sb = new StringBuilder();

String N = System.getProperty("line.separator");

String D = ";";

//default delimiter for .csv for excel

sb.append(getParameters());

sb.append(N);

sb.append("results:"+N+N);

sb.append("generation:"+D);

for(int i=0;i<data.length;i++){

sb.append(i+1+D);

}

sb.append(N);

sb.append("groups with divergent haplotypes ("+DISTANCE_OF_DIVERGENT_HAPLOTYPES+" differences constitute divergence):");

sb.append(D);

for(int j=0;j<data.length;j++){

sb.append((int)data[j][0]+D);

}

sb.append(N);

sb.append("groups with only similar haplotypes:");

sb.append(D);

for(int j=0;j<data.length;j++){

sb.append((int)data[j][1]+D);

}

sb.append(N);

sb.append("groups fixed at one haplotype:");

sb.append(D);

for(int j=0;j<data.length;j++){

sb.append((int)data[j][2]+D);

}

return sb.toString();

}

public String getDescriptionForHaplotypeDistance(double[][] data){

StringBuilder sb = new StringBuilder();

String N = System.getProperty("line.separator");

String D = ";";

//default delimiter for .csv for excel

sb.append(getParameters());

sb.append(N);

sb.append("greatest distance between haplotypes:"+N);

sb.append("generation:"+D);

for(int i=0;i<data.length;i++){

sb.append(i+1+D);

}

sb.append(N);

sb.append(D);

for(int i=0;i<data[0].length;i++){

for(int j=0;j<data.length;j++){

sb.append(data[j][i]+D);

}

sb.append(N+D);

}

return sb.toString();

}

public String getDescriptionForHaplotypePersistence(int[] data){

StringBuilder sb = new StringBuilder();

String N = System.getProperty("line.separator");

String D = ";";

sb.append(getParameters());

sb.append(N);

sb.append("Test for Haplotype Persistence"+N);

sb.append(data[0]+" haplotypes persisted past the cutoff generation (these are counted in the first field of the generation data below)"+N);

sb.append("Generation:"+D);

sb.append("(past cutoff)"+D);

for(int i=1;i<data.length;i++){

sb.append(i+D);

}

sb.append(N);

sb.append("Number of groups whose haplotype went extinct:"+D);

for(int i=0;i<data.length;i++){

sb.append(data[i]+D);

}

sb.append(N);

sb.append("Generations until extinction, for each group (each field represents one group, the number gives the generations until extinction):"+N);

sb.append(D);

for(int i=1;i<data.length;i++){//start at 1 to avoid the haplotypes at 0

//which are the ones that persisted past cutoff

for(int j=0;j<data[i];j++){

sb.append(i +D);//i is the number of the current generation

}

}

return sb.toString();

}

public static String format(double d){

//format a number so excel doesn't change it into a date, shift the period etc. ...

String s = String.valueOf(d);

StringBuffer sb = new StringBuffer();

for(int i=0;i<s.length();i++){

char c = s.charAt(i);

if(c == '.'){

sb.append(',');

}

else sb.append(c);

}

sb.append("00");//trailing zeros prevent it being read as a date

return sb.toString();

}

}
